# Definition, Epidemiology and Pathophysiology of Lymphoedema

**DOI:** 10.3390/cells14241955

**Published:** 2025-12-09

**Authors:** Erich Brenner, René Hägerling, Vivien Schacht, Klaus Schrader, Jörg Wilting

**Affiliations:** 1 Institute for Clinical and Functional Anatomy, Medical University of Innsbruck, Müllerstrasse 59, 6020 Innsbruck, Austria; erich.brenner@i-med.ac.at; 2 Research Group ‘Lymphovascular Medicine and Translational 3D-Histopathology’, Institute of Medical and Human Genetics, Charité-Universitätsmedizin Berlin, Augustenburger Platz 1, 13353 Berlin, Germany; rene.haegerling@charite.de; 3 Berlin Institute of Health at Charité-Universitätsmedizin Berlin, BIH Center for Regenerative Therapies, Augustenburger Platz 1, 13353 Berlin, Germany; 4 Department of Dermatology, Allergology and Venerology, Hannover Medical School, Carl-Neuberg-Strasse 1, 30625 Hannover, Germany; schacht.vivien@mh-hannover.de; 5 Praxis für Gefäßkrankheiten, Lilienthalstrasse 2, 95032 Hof, Germany; post@gefaesspraxis-hochfranken.de; 6 Department of Anatomy and Cell Biology, University Medical Center Göttingen, Kreuzbergring 36, 37075 Göttingen, Germany

**Keywords:** lymphoedema, staging, epidemiology, hypoxia, extracellular matrix, obesity

## Abstract

Lymphoedema is a physically and psychologically stressful, chronic progressive disease caused by long lasting damage or malfunction of the lymphatic drainage system. It is classified as primary when caused by a genetic predisposition (inherited or congenital) directly affecting any part of the lymphatic vascular system, or, much more often, as secondary (acquired) when caused by factors outside the lymphatic system, such as tumours or their treatment. As part of the development of an S3 guideline for the diagnosis and treatment of lymphoedema in German-speaking countries, we present here the definition of lymphoedema, its epidemiology, staging, pathophysiology, aggravating comorbidities, and differentiation from other forms of oedema or volume-increasing diseases. We refer to molecular links with obesity, present a diagram of possible pathomechanistic interactions, and finally discuss approaches for potential drug therapies. More intensive molecular genetic diagnostics of primary lymphedema seem to be gaining ground in Germany currently. We recommend further strengthening these diagnostics, as this is the only way to expand therapeutic options in the future and use existing therapies more efficiently.

This text is based upon and updates the basic text of the German S2k guideline ‘Diagnosis and Treatment of Lymphedema’ (AWMF, Version 3.0); dated 23 February 2017.

## 1. Definition of Lymphoedema

Lymphoedema is a physically and mentally stressful, chronically progressive disease affecting the interstitium, and based on primary (genetic) or secondary (acquired) damage to the lymphatic drainage system (Table 1), i.e., the initial lymph vessels (lymph capillaries, lymph sinuses), precollectors, lymph collectors, lymphatic trunks and/or lymph nodes; see also Ref. [1]. Under physiological conditions, there is an equilibrium between the fluid filtered through the blood vessel walls into the interstitium (lymphatic load) and its removal by the lymphatic vessel system (transport capacity of the lymphatic drainage system). Reabsorption of fluid into the venous branch of the blood capillaries and venules, postulated for many decades as Starling’s equilibrium, does not occur in most organs under physiological conditions [2,3,4]. Exceptions are the gastrointestinal tract, the kidneys and the lymph nodes. The lymphatic system is therefore quantitatively the decisive factor for interstitial drainage in most organs. Of the approximately 5 litres filtered from the blood vessel system per day in a 70 kg person, approximately 50% reaches the venous angle via the lymphatic trunks. Another 50% is reabsorbed into the blood vessels in the lymph nodes due to the oncotic pressure difference between lymph and blood. The protein content of serum (adults 6–8 g/dL) is about twice as high as that of lymph (adults 3–4 g/dL; exception: hepatic lymph 6 g/dL) [5,6]. See Section 4 for the protein content of lymphoedema fluid.

An insufficient lymphatic drainage system leads to quantitative and qualitative changes in the interstitial fluid. As the disease progresses, it is characterised by tissue alteration, regardless of its anatomical location, i.e., in all organs [7], most noticeably in the skin. Since lymphoedema (if not resulting from an obvious venous disease) is suspected of being malignant per se, the term ‘malignant lymphoedema’ should be mentioned here in particular. It should always be borne in mind that lymphatic drainage can be severely impeded by space-occupying compression of lymph vessels, by *lymphangiosis carcinomatosa* (tumour invasion of the lymphatics), or by lymph node involvement. The lymph then seeks the ‘path of least resistance’.

A genetic predisposition causes primary lymphoedema. It can occur as a single feature or as part of a complex syndrome. In addition to chromosomal disorders (e.g., Ullrich-Turner syndrome), the majority of primary lymphoedema cases are associated with pathogenic changes in individual genes. These can be inherited via the germ line, occur de novo in an affected individual, or sporadically affect individual regions of the body as part of a somatic mutation (somatic mosaicism). Depending on when the primary lymphoedema manifests, a distinction is made between congenital (manifestation of lymphoedema in or before the first year of life) and late-onset forms (late-onset lymphoedema) manifesting after the first year [8]. Several genes are known whose mutations can lead to lymphoedema, 19 of which are summarised in Table 2. In addition, lymphoedema is often part of syndromic diseases, such as Noonan syndrome. Lymphoedema can also occur in the context of overgrowth syndromes (e.g., overgrowth of the extremities) [9]. These include, in particular, syndromes associated with the RAS/MAPK/ERK or PI3K/AKT/mTOR signalling pathway. Traditionally, these diseases are/were named after their first describers (e.g., Klippel-Trénaunay-Weber syndrome, Turner syndrome). The genetic prerequisites are increasingly being studied. This has also led to the identification of the first gender-dependent forms of primary lymphoedema, e.g., for CELSR1 (cadherin EGF LAG seven-pass G-type receptor 1). Female carriers of a *CELSR1* mutation show earlier onset of the disease and complete or very high penetrance, while male carriers show incomplete penetrance and lymphoedema in less than 25% of cases (https://www.orpha.net/en/disease/detail/569816 (accessed on 13 October 2025)). A syndromic association has also been described for *CELSR1* [10].

Depending on the question, human genetic testing should be performed on a blood sample or a tissue sample from the affected region. Human genetic testing should be carried out at a Centre for Human Genetics specialising in lymphoedema and lymphatic vessel diseases and is available in German-speaking countries. (See: *The Human Genetics Quality Network*; https://www.hgqn.eu/ (accessed on 13 October 2025)).

Secondary lymphoedema can develop due to various diseases and injuries or as a result of therapeutic measures. Iatrogenic causes include, for example, the removal of axillary lymph nodes after breast cancer [29], but also the excision of pelvic, para-aortic, inguinal and femoral lymph nodes [30,31] as well as neck dissection, especially in combination with radiotherapy [32,33,34,35,36].

If left untreated, lymphoedema is characterised by a chronic, progressive course with consecutive augmentation and alteration of the interstitial tissue fluid and the extracellular matrix (formed and unformed components; the latter formerly referred to as ground substance). It should be noted that lymph is not only an ultrafiltrate of blood but also transports organ- and tissue-specific secreted proteins [37]. As the disease progresses, it is characterised by trophic disorders of tissues and organs, accompanied by an increase in connective or scar tissue (fibrosclerosis, especially after secondary infections) and fatty tissue, as well as quantitative and qualitative changes in the composition of the extracellular matrix (more and altered collagen types, decrease in elastin, increased glycosaminoglycans such as hyaluronan) [38,39]. The trophic disorders in the tissue apparently also result from a reduction in local O_2_ partial pressure and an increase in anaerobic metabolism [40]. Lymphatic endothelial cells, too, react to hypoxia with altered expression of genes that control the extracellular matrix [41,42].

The disrupted lymph flow also results in a disruption of leukocyte circulation. The lymph of the skin typically contains the following cell types: T cells: 80%, Langerhans (dendritic) cells: 6–10%, monocytes: 2–8%, B cells: 1–4%. Lymphoedematous tissue promotes acute infections (e.g., erysipelas, [in English often called cellulitis] as an acute infection caused mainly by haemolytic group A streptococci) [43,44]; see AWMF guideline: https://register.awmf.org/de/leitlinien/detail/013-100 (accessed 13 October 2025).

### Distinction from Other Forms of Oedema

Oedema is the accumulation of fluid in intra- or extracellular spaces. It can occur as a symptom of a variety of diseases or non-physiological stress and can be generalised or localised, painful or painless. It is most common in the lower extremities.

Causes of generalised oedema can include heart insufficiency, kidney disease, liver disease, myxoedema and pregnancy.

Causes of localised oedema can include venous insufficiency, thrombosis, intolerance reactions, inflammation and allergies, trauma, space-occupying processes and surgical procedures. Lymphoedema generally presents as a localised oedema caused by a functional impairment of the lymphatic system. Thereby, inflow from the blood vessels into the interstitium exceeds the transport via the lymph vessels [45].

Mixed forms with lymphoedema (mixed oedema) are frequently observed in clinical practice. For example, between 2005 and 2020 in Germany, lymphoedema was diagnosed in 0.9% of cases of deep vein thrombosis accompanied by oedema [46]. In contrast to oedema caused by arterial or venous causes, pure lymphoedema practically never causes ulcerations [47].

## 2. Epidemiology of Lymphoedema

The incidence of primary lymphoedema at birth is estimated to be approximately 1:6000 [48], with a prevalence of roughly 1:87,000 among people under the age of 20 [49]. Exact figures on the incidence of secondary lymphoedema are also challenging to determine, and the causes vary considerably worldwide. In industrialised countries, the incidence of secondary lymphoedema is estimated to be between 0.13 and 2%. The number of people affected in England (approx. 60 million inhabitants) in 2003 was reported to be 100,000 (0.17%) [50]. However, in English-speaking countries, the terms ‘lymphoedema’ and ‘chronic oedema’ are often used interchangeably, which makes precise recording difficult (https://www2.healthservice.hse.ie/organisation/national-pppgs/all-ireland-lymphoedema-guidelines/ (accessed on 13 October 2025)).

Women are significantly more affected by primary lymphoedema than men; m: f = 1:4.5 [50,51] to 1:6.1 [52]. Women are also more frequently affected by secondary lymphoedema [50]. The number of people affected increases with age. The most common cause of secondary lymphoedema is malignant tumours and their treatment. Lymph node removal in the inguinal region leads to lymphoedema more frequently than removal in the axillary region [50]. The data on the number of cancer patients who suffer from lymphoedema varies greatly [53]. However, thanks to improved surgical techniques, the situation has clearly improved in recent years. The incidence of lymphoedema 12–24 months after breast cancer is 19.9% after axillary lymph node removal and 5.6% after sentinel lymph node biopsy [54]. Since the individual number of lymph collectors and lymph nodes in a patient is not known, the following applies: Every lymph node that is spared counts! This recommendation is supported by the fact that axillary lymph nodes are increasingly being spared and not resected in certain minimal forms of breast cancer [55].

For gynaecological tumours, the incidence of lymphoedema after lymph node removal is reported to be approximately 20% [32,56,57]. However, some studies have found rates of 47% [58] and 60% [59] after specific gynaecological procedures. The highest risk of developing lymphoedema is associated with multimodal treatment consisting of chemotherapy, radiation, lymph node removal, as well as advanced stages of the disease and a higher body mass index [33]. Epidemiologically significant is the fact that obesity has an inducing and aggravating influence on lymphoedema [60,61].

In breast cancer, arm lymphoedema is most commonly documented. Figures on breast and chest wall oedema are less frequently recorded [62], although peritumoral oedema can obviously be observed in approximately half of patients. Thereby, residual breast oedema is a negative prognostic factor [63].

In recent years, there appears to be a trend towards improvement in the situation of fully hospitalised patients with ICD10:I89.0 ‘Lymphoedema, not classified elsewhere’ in Germany. Since 2016, the number has fallen steadily from 5368 (1563 male, 3805 female) to 3238 (1071 male, 2167 female) in 2023 (https://www.gbe-bund.de (accessed on 13 October 2025)). This corresponds to a reduction in the number of cases from 6 to 4 per 100,000 inhabitants. Most patients are over 65 years of age, and the average length of stay in hospital has remained the same at approximately 7 days (Supplementary Table S1).

However, the available data must be viewed in a differentiated manner:

- In 2017, differentiated ICD10 codes were introduced. This means that there are not only I89.0, Q82.0 and I97.2, but a large number of coding keys. It can be expected that these were also used.
- Hospitals often only treat patients with serious illnesses (heart insufficiency, COPD, diabetes mellitus with complications, extreme obesity) that make the outpatient phase 1 of complex physical decongestive therapy (CPT) impossible. It is therefore possible that lymphological codes play only a minor role (in terms of billing).
- It is possible that many patients have migrated to outpatient care.
- The length of stay of 7 days is difficult to understand, as successful lymphoedema reduction usually takes at least 12 days, see also Ref. [64].

A similar trend to I89.0 can also be seen in fully hospitalised patients with ICD10:I97.2 ‘Lymphoedema after partial mastectomy’. In 2016, there were 245 cases, and in 2023, there were 94 cases (Supplementary Table S2). However, the extent to which the data on inpatient cases reflect the outpatient situation remains to be discussed. Nevertheless, there have been clear signs of a positive development in recent years, and there is hope that diseases of the lymphatic system are becoming more widely recognised by doctors and patients.

Hereditary lymphoedema (Q82.0) was particularly common in 2010 and 2011, with 67 and 61 cases, respectively, and most recently down to 19 (2021) and 11 (2022) cases (Supplementary Table S2) (https://www.gbe-bund.de (accessed on 13 October 2025)). In 2023, a massive increase was noted (164 cases), most likely due to a significant expansion of molecular genetic diagnostics. We consider this trend to be positive. Genetic diagnostics will manifest itself in improved and safer therapy in the future.

## 3. Staging of Lymphoedema

Lymphoedema is often divided into ‘acute’ and ‘chronic’, although, however, there is no clear time frame. So-called acute lymphoedema, e.g., following injury to the lymph vessels or organ transplantation, resolves after a reasonable period of time, generally without permanent damage. The basis for this is the high regenerative capacity of the lymph vessels, including the collectors [65]. Only long-lasting lymphoedema leads to pathological changes in the affected tissues and organs. In English-speaking countries, lymphoedema is therefore often equivalent to ‘chronic oedema’ [66,67].

In accordance with the International Society of Lymphology, lymphedema is classified into three stages (I, II, III) and a subclinical stage, in which malfunctioning or damage to the lymph vessels is present but has not yet manifested clinically (Table 3) [1].

## 4. Pathophysiology of Lymphoedema

Lymphoedema is a condition that, in stages I and II, causes an accumulation of fluid in the interstitium, which is initially free and later becomes bound to ground substance (glycosaminoglycans).

There are two main mechanisms for the formation of interstitial oedema:

1. Pathologically high influx of fluid from the blood vessels into the interstitium;
2. Insufficient outflow of fluid via the blood or lymph vessels.

Traditionally, a distinction is made between protein-poor and protein-rich oedema, with lymphoedema being classified as protein-rich. However, the original definition of ‘protein-rich’ is often unknown or not specified. The definition appears to be inaccurate and should be questioned, as the protein content of arm lymphoedema, for example, is even lower than that of comparable interstitial arm fluid on the healthy side [68]. One hypothesis could be that increased capillary pressure with an intact protein barrier in the oedematous arm leads to an increased filtration rate. Intercellular fluid exhibits organ-specific differences, which must always be considered in such comparisons. Another hypothesis relates to proteoglycans, which are broken down less efficiently in lymphoedema and therefore bind more fluid [69]. These proteoglycans exert a strong colloid osmotic pressure, as do the proteins in the extracellular matrix.

The boundary between ‘protein-rich’ and ‘protein-poor’ was originally defined at 1 g/dL [70], as cardiac, venous, and hypoproteinaemic oedema are below 1 g/dL, while lymphoedema is around 3 g/dL [71,72]. The protein content of lymphoedema may therefore be considered to be ‘normal’. A higher protein content of 4–6 g/dL is found in inflammatory, allergic and burn oedema [71]. In these forms of oedema, the barrier function of the endothelial cells of the blood vessels is defective, so that these oedemas could be described as ‘protein-rich’.

However, as early as the 1950s, it was suggested that the progression of primary leg lymphoedema was characterised by an accumulation of interstitial proteins. However, the mean values for ‘moderate’ and ‘severe’ lymphoedema were 2.5 g/dL vs. 3.4 g/dL, respectively, which were always within the normal range, and no comparison with a potential control side was made [72].

Elevated colloid osmotic pressure (indicating an increase in protein or glycosaminoglycan concentrations) has been measured in the interstitium in primary lymphoedema in animals [73]. See below for hyaluronan.

Lymphoedema is characterised by a typical sequence of tissue changes that occur mainly in the intercellular space and in the cells that control this space. However, only preliminary studies have investigated which cells and molecules regulate the subsequent changes in the interstitium.

### 4.1. Changes in Cells and the Intercellular Space in Lymphoedema

When lymphoedema occurs in the skin, the following changes can be observed: In the initial stage (stage I), an increase in free interstitial fluid is measurable. In advanced stages (stages II, III), the following clinical symptoms occur:

1. Thickening of the cutis and subcutis due to
  - Accumulation of adipose tissue;
  - Reactive proliferation of connective tissue (due to hypoxia or weight; fibrosis and sclerosis also occur after erysipelas);
  - Formation of lymph cysts and fistulas; chylous cysts and fistulas in rarer cases of central drainage disorders.
2. Trophic changes in the epidermis and dermis.
  - Hyperplasia with hyperkeratosis;
  - Mild papillomatosis;
  - Extensive node and nodule formation (the term “elephant skin” is no longer appropriate);
  - Discolouration (hyperpigmentation), usually only in combination with blood vessel disorders.
3. Disorders of the local immune defence.
  - Susceptibility to erysipelas;
  - Susceptibility to fungal infections;
  - Other.
4. Painful changes in the musculoskeletal system.

#### 4.1.1. Changes in the Intercellular Space

**Figure 1:**
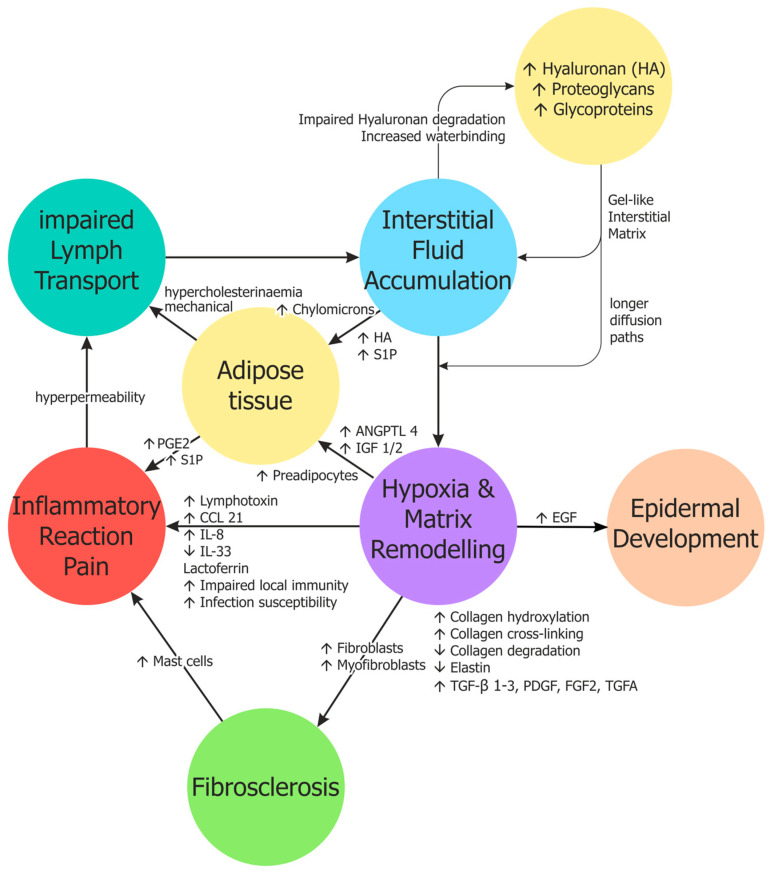
Schematic representation of the pathophysiology of lymphedema. For further details, see text. ANGPTL4—Angiopoietin-like 4; CCL21—C-C motif chemokine ligand 21; EGF—Epidermal growth factor; FGF2—Fibroblast growth factor 2; IGF-1/2—Insulin-like growth factor-1/2; IL-8/33—Interleukin 8/33; PGE2—Prostaglandin E2; S1P—Sphingosine-1-phosphate; TGA—Transforming growth factor-α; TGF-β—Transforming growth factor-β.

The molecular changes in the intercellular space have not been adequately studied in lymphoedema. In the early stages, the tissue is characterised by increased transparency and reduced histological dyeability [39], presumably based on increased storage of water-binding hyaluronan [38], which cannot be visualised in routine histological staining. As a non-sulphated glycosaminoglycan consisting of up to 50,000 repetitive disaccharides, hyaluronan has enormous water-binding potential [6,74], and creates a gel. It is produced by many different cells, most effectively by smooth muscle cells and fibroblasts. Approximately 90% of its degradation occurs in the lymphatic endothelium, with the remaining 10% entering the blood via the efferent lymph and being broken down in the liver sinusoids. The lymphatic and sinus endothelial cells possess the specific hyaluronan receptor LYVE-1 for this purpose [75]. Low-molecular-weight hyaluronan is excreted via the kidneys. It can be assumed that the breakdown and removal of hyaluronan and its metabolites is severely impaired in lymphoedema. However, the use of hyaluronidase for the treatment of lymphoedema has not yet been sufficiently investigated [76,77]. The accumulation of hyaluronan is a key factor in the pathophysiological progression of lymphedema, which we have summarised schematically in Figure 1.

In stages II and III, an increase in fibrous material in the interstitium can occur [39]. Type I and type III collagen have been detected immunohistochemically. However, other collagen types are also present. The thickness of the corium thus increases significantly. There are areas in which the fibrous material is clearly visible, while in other areas, amorphous substances (including fibronectin and albumin—but otherwise largely unidentified) predominate. The diameter of the collagen fibres increases to 40–400 nm compared to 25–200 nm in normal skin, and there is an increase in ‘long-spacing’ collagen, which has a periodicity of 80–120 nm (instead of 64 nm) in its cross-striation [78]. Changes in the basement membranes (discontinuities) are detectable at the electron microscopic level. The basement membranes have often detached from the endothelium of the blood vessels. A dense matrix resembling a basal lamina develops around the initial lymphatic vessels. It is not clear whether there is a change in the elastic fibre networks and the anchoring filaments. However, lymphatic endothelial cells cultivated in vitro under hypoxia (comparable to a hypoxic situation in lymphoedema) show increased activity of collagen hydroxylating enzymes (PLOD2, P4HA1), collagen cross-linking enzymes (LOX), collagen degradation-inhibiting enzymes (ITIH5), and reduced elastin mRNA expression [41,42]. ITIH5 not only inhibits the degradation of collagen but also of hyaluronan, to which it is covalently bound. It is also overexpressed in obesity, which could explain the lymphoedema-aggravating influence of obesity [79]. Reduced elastin levels have been observed in lymphoedema, particularly in the initial lymphatics [80]. In addition to muscle hyperplasia, an increase in fibre content has been observed in the lymph collectors [39].

Matrix metalloproteinases (MMPs), which break down the various matrix components, have been described immunohistologically in lymphoedema. However, their activity has not yet been investigated. However, under hypoxia in lymphatic endothelial cells there is a significant downregulation of mRNA of one of these enzymes (ADAMTS15), which suggests a stabilisation of the matrix [6,41]. Type IV collagen is significantly reduced in the serum of patients with primary and secondary lymphoedema [6]. MMP1, which is upregulated under hypoxia, is a double-edged sword, as it can induce not only matrix degradation but also fibrosis [81]. One reason for this could be that inactive growth factors bound to the matrix are released and activated by matrix digestion, including transforming growth factor-β (TGFβ) [82].

The changes in the extracellular matrix could be controlled by TGF-β, which is secreted by dendritic cells, among others. TGF-β could act in two ways: as an inhibitor of lymphangiogenesis and as the primary activator of scar formation and fibrosis [83]. TGF-β2 and TGF-β3 are significantly upregulated in the lymphatic endothelium under hypoxia [6,41].

An interaction between lymphatic endothelial cells and the extracellular matrix is also possible at the level of coagulation factors, albeit significantly reduced as compared to the blood endothelium. Lymph contains fibrinogen [37]. Lymphatic endothelial cells are the main producers of factor VIII alongside the liver, and they also produce its carrier protein, von Willebrand factor [84]. A link between thrombosis and lymphoedema has been described [85,86]. However, it should be noted that lymphatic endothelial cells have anticoagulant activity, e.g., through the high expression of ecto-5′-nucleotidase (CD73) [87]. CD73 is part of the enzymatic cascade by which platelet-aggregating extracellular ADP is degraded [88].

#### 4.1.2. Changes at the Cellular Level

In the initial stage of lymphoedema, molecular changes in the local cells (lymphatic endothelial cells, smooth muscle cells, preadipocytes, fibrocytes, resident macrophages and mast cells) are likely to be prominent. Mast cells are regularly found in the wall of lymph collectors [89]. In stages II and III, a general increase in the number of cells can be observed [39]. In addition to the massive development of adipocytes, the proliferation of fibroblasts is typical, whose transdifferentiation into myofibroblasts is controversially discussed [39,78]. Cells that have been interpreted as pericytes are found at the initial lymph vessels [78], but the lymphatic endothelial cells themselves may also change under hypoxia and contribute to fibrosis (see above, Section 4.1.1).

CD68-positive macrophages are frequently observed, apparently phagocytosing collagen fibres. The number of CD34-positive cells is large [39]. This transmembrane glycoprotein is expressed in a subpopulation of dendritic cells, as well as in hematopoietic precursor cells, endothelial cells of blood vessels and mast cells. The number of dendritic cells that are factor XIIIa-positive is significantly increased. Both the number of immigrated and resident macrophages/antigen-presenting cells is thus increased. The resident macrophages proliferate in a phase described as the pre-chronic phase. These cells produce a wide range of cytokines, chemokines, and growth factors. However, no signs of granulomatous inflammation can be observed [39].

Some chemokines are also produced by lymphatic endothelial cells. Among these is interleukin 33 (IL33), which is significantly down-regulated under hypoxia in lymphatic endothelial cells [6]. IL33 is also known as ‘alarmin’ [90]. It binds to ST2 (suppression of tumorigenicity 2) receptors on mast cells, Th2 helper cells, and type 2 innate lymphoid cells (ILCs) [91]. Th2 cells secrete IL4 and IL5, and thereby induce an isotype-switch of B cells, so that more effective, smaller immunoglobulins are produced. Additionally, eosinophilic granulocytes are activated for effective defence against parasites and fungi. Due to the disrupted skin barrier, there is a high risk of fungal infection in lymphoedema [92]. In obesity, soluble ST2 receptors have been found that inhibit IL33 function [91], which, together with hypoxia-induced down-regulation of IL33 in lymphatics, might explain increased susceptibility for infection and inflammation in lymphoedema.

The molecular factors that control the massive development of adipose tissue in lymphostasis have not been investigated in detail. Redistribution of adipose tissue, as observed in Crohn’s disease and HIV, suggests possible interactions between adipose tissue and the immune system [93]. The lipid sphingosine-1-phosphate (S1P) could play an important role in this process. S1P is found in all body fluids, but is highest in lymph [94]. As a chemotactic lipid, it attracts lymphocytes from the lymphatic organs into the lymph [95]. S1P binds to specific surface receptors and can stimulate the expression of cyclooxygenase 2 (prostaglandin synthase 2) and the secretion of prostaglandin E2 [96], which contributes to inflammation and pain sensitization. S1P is also elevated in obesity [97].

Both S1P and hyaluronan are high-calorie molecules that apparently accumulate in the tissue in lymphoedema. The development of adipose tissue is known to depend on calorie intake. Visceral adipose tissue also develops, for example, when chylomicrons leak from the intestinal lymph vessels [98]. Obviously, local calorie supply can promote the development of adipose tissue, especially since preadipocytes are ubiquitously present in loose connective tissue.

The number of lymphatic vessels in the various stages of lymphoedema has not yet been systematically investigated. Only animal experiments are available, but these date from a time when immunohistological identification of lymphatic vessels was not yet possible [99]. Whether this leads to the reported relative increase in volume and length of the initial lymph vessels requires further investigation. The upregulation of the lymphangiogenic growth factor VEGFC in hypoxic lymphatic endothelial cells suggests that the expansion of the initial lymphatics in lymphoedema is due to lymphangiogenesis in addition to congestion [6]. There are reports that lymph collectors react with muscle hypertrophy and fibrosis [100]. There are also indications of fibrosis of lymph nodes and afferent lymph collectors in advanced lymphoedema [39].

## 5. Molecular Basis of Primary Lymphoedema

Primary lymphoedema probably accounts for only about 1% of all cases of lymphoedema [101,102,103], but the absolute number of cases worldwide is likely to be very high. For affected patients (who wish to have children), it will not only be of interest to know how the underlying genetic defect is inherited, but also whether there is a specific, causal treatment. In addition, knowledge of the causal genetic alteration plays an important role in the diagnosis and prevention of concomitant diseases (e.g., acute myeloid leukaemia in Emberger syndrome). Human genetic diagnosis and counselling for patients are highly desirable. Causal therapies cannot yet be offered, but cannot be ruled out in the future. Factors that control the structure and function of lymphatic vessels are already known, and initial investigations into pharmacological interventions based on precision medicine principles are already underway.

Depending on the issue at hand, genetic diagnostics should include basic diagnostics (chromosome analysis and array CGH) as well as molecular genetic methods such as whole-exome or whole-genome sequencing. This allows all genes associated with primary lymphoedema to be analysed. These include the following genes in particular (see also Table 2) [104]. Further genes will be added in the future.

### 5.1. Primary Lymphoedema with Myelodysplasia (Emberger Syndrome)

Gene: *GATA2*; OMIM No.: 614038. The *GATA2* gene is located on chromosome 3q21.3. It encodes a transcription factor that, in addition to blood cell development, also appears to control the function of lymph vessels in an unknown manner. Emberger syndrome is often characterised by congenital facial dysplasia. In childhood or early adulthood, patients develop leg lymphoedema, often accompanied by genital lymphoedema, pancytopenia or acute myeloblastic leukaemia (AML) [11,105].

### 5.2. Microcephaly with or Without Chorioretinopathy, Lymphoedema or Intellectual Disability

Gene: *KIF11*; OMIM No.: 152950. The *KIF11* gene is located on chromosome 10q23.33 and encodes a kinesin family motor protein. *KIF11* (the protein is called Eg5) is essential for the transport of chromosomes along the microtubules of the mitotic spindle during cell division [106]. Eg5 is highly expressed in blood and lymphatic endothelial cells and regulates angiogenesis [107]. Complete loss of *KIF11* function is incompatible with life [108]. Mutations in the KIF11 gene cause a variety of serious defects and lymphoedema on the back of the foot, which are present at birth or shortly thereafter [12]. However, eating and growth disorders in children and unpublished findings suggest that the lymph vessels of the gastrointestinal tract are also affected, so that chylomicrons are probably not adequately removed (https://kif11kids.com (accessed on 13 October 2025)).

### 5.3. Oculo-Dento-Digital Dysplasia

Oculo-dento-digital dysplasia (OMIM #164200) is characterised by a phenotype that primarily affects the eyes (oculo), teeth (dento) and fingers (digital). Typical features include microphthalmia and other eye abnormalities, small or missing teeth and weak tooth enamel. Camptodactyly or syndactyly between the fourth and fifth fingers, and occasionally between the toes, is also common. Primary lymphoedema is usually present as well. Changes in the *GJA1* gene have been identified as the cause. *GJA1* encodes connexin 43, a protein that plays a central role in gap junction formation. Gap junctions play an essential role in cell–cell communication and have been described in various cell types, including the lymphatic vessel valves [109].

### 5.4. Hennekam Syndrome

Hennekam syndrome is a progressive congenital primary lymphoedema that affects not only the extremities but the entire body. It is characterised by specific facial dysmorphic features, often accompanied by systemic manifestations such as hydrops fetalis, intestinal lymphangiectasia, chylothorax, and ascites. Hennekam syndrome is caused by genetic changes in *CCBE1* (Hennekam syndrome type 1, OMIM #235510), *FAT4* (Hennekam syndrome type 2, OMIM #616006) or *ADAMTS3* (Hennekam syndrome type 3, OMIM #618154) [109]. Mutations in the *CCBE1* and *ADAMTS3* genes reduce processing of the lymphatic growth factor VEGF-C, leading to malformation of the lymphatics during embryonic development. In comparison, a pathogenic mutation in the *FAT4* gene leads to dysfunctional development of the lymphatic vessel valves. This explains the phenotypic differences between the respective subtypes [109].

### 5.5. Generalised Lymphatic Dysplasia According to Fotiou

Generalised lymphatic dysplasia, according to Fotiou (OMIM #616843), shows a similar phenotype to Hennekam syndrome, but the primary lymphoedema is less pronounced. It is caused by pathogenic variants in the PIEZO1 gene, which encodes a mechanosensitive ion channel essential for the transmission of mechanical signals during lymphangiogenesis [16,109].

### 5.6. Lymphatic-Related Hydrops Fetalis (LRHF) and Lymphoedema

Gene: *EPHB4*; OMIM No.: 617300. In contrast to somatic mosaic mutations in *EPHB4* causing capillary and/or arteriovenous malformations, pathogenic germline variants cause an autosomal dominant disorder with variable expressivity. Some affected individuals may develop severe non-immune lymphatic-related hydrops fetalis (LRHF) during foetal development, which can result in prenatal death. In contrast, others present with milder symptoms, such as an atrial septal defect or varicose veins, which appear in adulthood. In those who survive the neonatal period, the hydrops and/or swelling improve spontaneously [110].

### 5.7. Hypotrichosis, Lymphoedema, Telangiectasia Syndrome (HLTS)

Gene: *SOX18*; OMIM No.: 607823. The SOX18 gene is located on chromosome 20q13.33 and encodes a DNA-binding transcription factor from the SRY family. HLTS is a complex disorder characterised by early hair loss, ectatic blood vessels, and lymphoedema. Mutations in the *SOX18* gene have been found in three HLTS families [18]. Those affected developed leg lymphoedema at the age of 15 or as early as 4 years [110].

### 5.8. Choanal Atresia and Lymphoedema

Gene: *PTPN14*; OMIM No.: 613611. The *PTPN14* gene is located on chromosome 1q41 and encodes a non-receptor-type protein tyrosine phosphatase. Protein tyrosine phosphatases catalyse the removal of phosphate groups from phosphorylated proteins. PTPN14 binds to VEGFR-3 and reportedly reduces its activity [111]. Mutations that inactivate PTPN14 catalytic activity cause leg lymphoedema and choanal atresia in childhood [19].

### 5.9. Hereditary Lymphoedema I-A (Nonne-Milroy Lymphoedema)

Gene: *FLT4* = *VEGFR3*; OMIM (Online Mendalian Inheritance in Man) No.: 153100. The *FLT4* gene is located on chromosome 5q35.3 and encodes vascular endothelial growth factor receptor-3 (VEGFR-3). Heterozygous mutations in the *VEGFR3* gene are thought to be responsible for the majority of primary lymphoedema cases [112]. The mutations become apparent whenever they impair the function of the tyrosine kinase domain of the receptor [25,113,114].

### 5.10. Hereditary Lymphoedema I-D

Gene: *VEGFC*; OMIM No.: 615907. The *VEGFC* gene is located on chromosome 4q34.3. VEGF-C is the most important lymphangiogenic growth factor [115]. Mutations that impair the secretion and receptor affinity of the factor cause a Milroy-like disease with congenital or early childhood leg lymphoedema [116].

### 5.11. Lymphoedema-Distichiasis Syndrome

Gene: *FOXC2*; OMIM No.: 153400. The *FOXC2* gene is located on chromosome 16q24.1 and encodes the transcription factor FOXC2. Transcription factors regulate the expression of a large number of genes. Mutations in the important transcription activation domain of FOXC2 cause leg lymphoedema and, at the same time, the development of additional or double rows of eyelashes (distichiasis) [24]. In addition to leg lymphoedema, reflux into the *great saphenous vein* and the formation of varicose veins can also be observed in many patients, as FOXC2 regulates the development of valves in both the lymph collectors and the veins [117].

### 5.12. Hereditary Lymphoedema I-C

Gene: *GJC2*; OMIM No.: 613480. The *GJC2* gene is located on chromosome 1q42.13 and encodes the gap junction protein gamma 2 (connexin 47; CX47). Connexins are transmembrane proteins that connect two cells in the form of connexons (gap junctions) as hemi-channels, thereby enabling the exchange of ions and low-molecular substances. Mutations in the *GJC2* gene have been identified in several families with arm and leg lymphoedema (onset of oedema between the ages of 1 and 40) [25,118].

### 5.13. CELSR1-Related Lymphoedema

The *CELSR1* gene plays a key role in the planar cell polarity signalling pathway. Changes in CELSR1 are associated with primary bilateral late-onset lymphoedema of the lower extremities, and it is potentially the gene causing Phelan-McDermin syndrome. Renal defects have also been described, so a nephrological examination is recommended for affected individuals. Inheritance is autosomal dominant with variable expressivity and incomplete penetrance, varying from 91% in female carriers to 23% in males [109].

### 5.14. Primary Lymphoedema Associated with HGF

Gene: HGF (Hepatocyte Growth Factor); OMIM No.: [not yet assigned]. The HGF gene encodes a growth factor that signals via the MET receptor and plays a crucial role in lymphatic vessel development and maintenance. Rare pathogenic variants in HGF explain about 2% of primary lymphoedema cases. Clinically, HGF-associated lymphoedema is usually bilateral in the lower limbs with variable onset from childhood to adulthood. Loss-of-function variants cause reduced HGF secretion and impaired activation of downstream PI3K/AKT and ERK1/2 pathways [26].

### 5.15. Primary Lymphoedema Associated with TIE1

Gene: *TIE1*; OMIM No.: 619401. The *TIE1* gene encodes an endothelial receptor tyrosine kinase that functions within the ANGPT/TIE signalling pathway, crucial for lymphatic vessel integrity. Rare loss-of-function variants in *TIE1* have been identified in patients with late-onset primary lymphoedema, causing impaired lymphatic endothelial signalling and vessel maintenance [27].

### 5.16. Primary Lymphoedema Associated with ERG

Gene: *ERG*; OMIM No.: 620602. The *ERG* gene encodes a transcription factor of the ETS family that is essential for blood and lymphatic vessel development and maintenance. Pathogenic variants in ERG cause primary lymphoedema affecting the lower and/or upper extremities, genitalia, and sometimes the intestinal tract (lymphangiectasia). Additional features may include varicose veins and cardiovascular abnormalities [28].

### 5.17. Noonan Syndrome

Noonan syndrome (OMIM #163950) is an example of primary lymphoedema that occurs in the context of syndromic diseases. Postnatal short stature, characteristic facial dysmorphic features, congenital heart defects and, in some cases, developmental delays characterise it. Noonan syndrome is one of the RASopathy syndromes, which are caused by mutations in genes of the RAS signalling pathway [109].

## 6. Differentiation from Obesity and Lipoedema

### 6.1. Obesity

Obesity is a major risk factor for the development of secondary lymphoedema, presumably due to mechanical obstruction of lymph transport [29,119,120], or due to molecular interactions (see above). Obesity is a consequence of excessive accumulation of triglycerides (fat) in fat storage cells (adipocytes). These cells are mainly located in the subcutaneous tissue, in addition to the internal organs (visceral fat). The body mass index (BMI) is used as a basis for calculation. It is calculated from body mass (m; given in kg) divided by height^2^ (in metres); BMI = kg/m^2^. Other significant factors are age, gender and fat distribution. Alternatively, the waist-to-height ratio can also be applied [121].

See also the AWMF guidelines:

- Therapy and prevention of obesity in children and adolescents (http://www.awmf.org/leitlinien/detail/ll/050-002.html (accessed on 18 November 2025))
- Surgery for obesity and metabolic diseases (http://www.awmf.org/leitlinien/detail/ll/088-001.html (accessed on 18 November 2025))

If lymphoedema accompanies obesity, lymphological considerations apply. However, lymphoedema is usually to be regarded as secondary and obesity as primary [122]. Conversely, lymphoedema can also lead to lymphoedema-associated obesity [123,124]. Obesity-associated lymphoedema is a form of secondary lymphoedema that can occur when a person’s BMI exceeds 40 kg/m^2^. The risk of lymphatic dysfunction increases with a higher BMI and is almost universal at a BMI of 60 kg/m^2^.

### 6.2. Lipoedema

Lipoedema is a symmetrical, pressure- and pain-sensitive accumulation of adipose tissue, almost exclusively in women, usually located below the iliac crest (hips, thighs) [125]. The lymphatic system is not primarily affected. For a definition of lipoedema, see AWMF guideline 037-012 [126]; (https://www.awmf.org/service/awmf-aktuell/lipoedem (accessed on 18 November 2025)).

The aetiology of lipoedema is unclear. A hereditary component is assumed, but it is very complex and cannot yet be narrowed down to a specific gene pool [127]. It is a distribution disorder of fatty tissue that, in most cases, affects women. The hormonal component of the disease is evident by the fact that it usually occurs at the onset of puberty, sometimes after pregnancy or during menopause. In men, lipoedema only occurs in cases of severe hormonal disorders [128]. In more severe cases of lipoedema, there is co-occurring obesity, which in turn can cause obesity-associated lymphoedema [129].

## 7. Conclusions

Lymphoedema that is not treated inevitably takes a chronic progressive course. Treatment is therefore strongly recommended. Patients who are at risk of developing lymphedema, e.g., after lymph node resection, should be strongly advised to take prophylactic measures. Prophylactic measures should also be started as early as possible in children who have a genetic predisposition to lymphoedema. Conservative treatment using complete decongestive therapy (CDT) is the first line of treatment. Even though CDT achieves outstanding results, a cure is not possible in many cases because the insufficient lymphatic drainage system cannot be completely restored or healed. Microsurgery can be an additional measure to restore a functioning lymphatic system. There is extensive literature on the diagnosis and treatment of lymphoedema [70,130].

Further measures for treating lymphoedema are currently experimental or theoretical. Medicinal approaches have only been pursued in very isolated cases. However, such measures could become increasingly important, as the prevalence of lymphedema could be much higher than previously thought. Since hyaluronan accumulates in stage-I lymphedema, the application of hyaluronidase could release bound water, making it accessible for displacement by CDT [76,77]. The use of indocyanine green (ICG) to locate functional collateral circulation could be helpful in the practical implementation of lymphatic drainage by physical therapists [131,132]. Additionally, the use of artificial drainage systems (silicone tubes) without [133] or with electric pumps has been discussed for some time [134].

During the progression into stages II and III, lymphoedema is accompanied by fibrosclerosis and adipose tissue development. Medication to support CDT, such as antifibrotic measures or the reduction in fat cells, would be feasible and desirable. Standard weight loss measures are, of course, part of the current therapy. Antifibrotic drugs will become increasingly important in medicine, not only for pulmonary fibrosis. The most potent inhibitors to date target typical growth factors that promote fibrosis: TGFβ and platelet-derived growth factor (PDGF). As discussed above for TGFβ, these factors are upregulated under hypoxia, not only in lymphatic endothelial cells, but also in fibrocytes and other cells [135]. The central intracellular regulator of hypoxic responses is the transcription factor Hypoxia-inducible factor 1 (HIF1), which not only controls angiogenesis and erythropoiesis but also multiple mechanisms and factors that augment cell growth and extracellular matrix production. Among these are TGFβ, PDGF-B, Fibroblast growth factor-2 (FGF2), Insulin-like growth factor-1 and -2 (IGF1/2), Epidermal growth factor (EGF), and Transforming growth factor-α (TGFA) (Figure 1). HIF1 not only targets protein-coding genes, but also long noncoding (ln)RNAs and miRNAs [136]. This could lead to further targets for antifibrotic therapy. Targeting the lnRNA H19 has already been tested in renal fibrosis [137].

The mechanisms of obesity that exacerbate lymphedema are also likely to be found at the molecular level. We have already mentioned some of the possible interactions above. Adipocyte progenitor cells are present in all connective tissues, and high calorie molecules can stimulate their differentiation, as has been shown for chylomicrons (review see: [6]). IGF1, a HIF1-regulated core-gene, induces differentiation of preadipocytes [138]. Local development of adipose tissue may also be caused by hypoxia-induced upregulation of Angiopoietin-like 4 (ANGPTL4) in lymphatic endothelial cells [42]. ANGPTL4 is a secreted inhibitor of lipoprotein lipase (LPL), which is produced and secreted by adipocytes [139]. LPL hydrolyses triglycerides (TGs). ANGPTL4 will increase local TG levels and may thereby stimulate adipose tissue development (Figure 1).

Obesity impairs lymph transport in lymph collectors. This may simply be a mechanical effect caused by kinking of collectors, particularly in overhanging skin folds. However, there is also evidence of molecular interactions that weaken the contractility of the collectors [140]. Powerful autonomic peristaltic waves of lymph collectors are the main driving force for transporting lymph against gravity and orthostasis from the foot to the venous angle in the neck. Peristalsis requires a regulated sequence of contraction and dilation, which can be observed in vivo using ICG [141]. Medication-induced enhancement of peristalsis could be significant in older people. There are several potential molecular targets for increasing the activity of smooth muscle cells or pacemaker cells in lymph collectors [89,142].

In principle, there are two ways to restore a damaged or hypoplastic lymphatic system: using the lymphangiogenic growth factor VEGF-C or using autologous induced pluripotent stem cells (iPSCs). Gene therapy using an adeno-associated VEGF-C expression vector has been performed in phase I and phase II studies. Thereby, autologous vascularized lymph nodes were transfected and transferred into the axilla of breast cancer patients with arm lymphoedema [143]. A more extensive comparison with other treatment methods is certainly still needed, and, importantly, much experience must first be gained before it can be determined whether the technique can be used to treat primary lymphoedema caused by VEGF-C deficiency.

Since the discovery of embryonic reprogramming of adult cells into iPSCs [144] and their subsequent differentiation into various cell types, there is great hope that organ defects can be repaired using autologous iPSCs. Differentiation of lymphatic endothelial cells from iPSCs has been reported [145,146], and it will be of interest to follow its clinical application in the future. And finally, in patients with lymphedema, care should always be taken to ensure that conditions that increase the lymphatic load—e.g., other types of oedema, or varicose veins—are treated immediately.

## Figures and Tables

**Table 1:** Causes of primary and secondary lymphoedema.

Primary	Secondary
Aplasia/atresia Hypoplasia Hyperplasia/dysplasia Lymphatic valve defects Lymph node fibrosis Lymph node agenesis	Malignant processes Traumatic/post-traumatic (scars) (Post-)infectious Surgical procedures Lymph node dissection Radiation Advanced stages of chronic venous insufficiency (CVI) Obesity Constrictions (amniotic band, artificial)

**Table 2:** Genetic causes of primary lymphoedema and clinical pictures.

Gene	Clinical Name		OMIM Number	Clinical Feature		Function of the Gene Product
*GATA2*	Emberger syndrome		614038	Syndromic diseases (group 2a)	- Lymphoedema of the lower extremities and genitals - Myelodysplasia to AML	Transcription factor expressed in hematopoietic stem cells and endothelial cells [11]
*KIF11*	Microcephaly-chorioretinopathy-lymphoedema syndrome		152950		- Lymphoedema of the lower extremities - Intellectual disability - Chorioretinopathy	Motor protein that plays a role in mitosis and vesicle transport [12]
*GJA1*	Oculo-dento-digital syndrome		164200		- Lymphoedema - Microphthalmia - Small or missing teeth - Weak tooth enamel - Camptodactyly - Syndactyly between the fourth and fifth fingers (possibly also the toes)	Connexin 43 as a component of gap junctions in lymphatic vessel valves plays a role in their correct development [13]
*CCBE1*	Hennekam syndrome	Type 1	235510	Lymphoedema with systemic involvement (group 2b)	- Progressive, congenital primary lymphoedema affecting the entire body - Intestinal lymphangiectasia - Hydrops fetalis - Chylothorax - Ascites - Facial anomalies	Role in the processing of VEGF-C (lymphatic growth factor) and the migration and proliferation of lymphatic endothelial cells (LECs) [14]
*ADAMTS3*		Type 3	618154			
*FAT4*		Type 2	616006			Role in the formation of lymph vessels [15]
*PIEZO1*	Generalised lymphatic dysplasia according to Fotiou		616843		- Similar to Hennekam syndrome, but milder	Mechanosensitive ion channel with a role in lymphangio- genesis [16]
*EPHB4*	Lymphatic-associated foetal hydrops (LRFH)		617300		- Hydrops fetalis - Atrial septal defect - Varicose veins - Lymphoedema of the lower extremities	Role in lymphatic vessel remodelling and valve development [17]
*SOX18*	Hypotrichosis-lymphoedema-telangiectasia-(renal) syndrome		607823		- Lymphoedema of the lower extremities - Hydrops fetalis - Hydrocele - Telangiectasia - Possible renal involvement	Induces expression of the lymphatic master transcription factor PROX-1 for development of LECs from venous endothelial cells [18]
*PTPN14*	Choanal atresia-lymphoedema		613611		- Bilateral choanal atresia - Lymphoedema of the lower extremity	Role in regulation of lymphangiogenesis and development of the choanes [19]
*DCHS1*	Van Maldergem syndrome		601390		- Overlapping clinical features and pathophysiology with Hennekam syndrome, but lymphoedema is rare in Van Maldergem syndrome	*FAT4* and *DCHS1* together play a role in the development of the lymphatic system and valve formation [20]
*FLT4*	Milroy disease		153100	Congenital lymphoedema (group 2c)	- Lymphoedema of the lower extremities (particularly the back of the foot and below the knee) - Varicose veins - Positive family history	*FLT4* codes for vascular endothelial growth factor receptor 3 (VEGFR-3), which is expressed on LECs and binds to VEGF-C, an important mediator of lymphangiogenesis [21,22]
*VEGFC*	Milroy-like disease		615907			
*FOXC2*	Lymphoedema-distichiasis syndrome		153400	Late-onset lymphoedema (group 2d)	- Distichiasis - Lymphoedema of the lower extremities - Varicose veins	Transcription factor that plays a role in the development of valves in veins and lymph vessels [23,24]
*GJC2*	Four-limb lymphoedema		613480		- Lymphoedema of the lower extremity, upper extremity and genitals	Connexin 47, as a component of gap junctions in lymphatic vessel valves, plays a role in their correct formation [25]
*CELSR1*	Phelan-McDermid syndrome		619319		- Lymphoedema of the lower limbs - Renal defects - Incomplete penetrance (women > men)	*CELSR1* gene product plays a key role in the planar cell polarity signalling pathway
*HGF*					- Lymphoedema of lower limbs; late onset	Hepatocyte growth factor activates AKT and ERG [26]
*TIE1*			619401		- Lymphoedema of lower extremity	TIE1 is a receptor tyrosine kinase regulating (lymph-) angiogenesis and vascular remodelling [27]
*ERG*			620602		- Lymphoedema of lower extremity, upper extremity, genitalia and/or intestinal lymphangiectasia - Varicose veins - Cardiovascular problems	ERG is a transcription factor of the Erythroblast Transformation Specific (ETS)-family, relevant for blood and lymphatic vessel development and maintenance [28]

**Table 3:** The stages of lymphoedema and clinical symptoms.

Stage 0 Latency stage Subclinical stage	No clinically apparent lymphoedema, but some feeling of heaviness (tension) and, in some cases, pathological lymphoscintigraphy
Stage I (spontaneously reversible)	Oedema of soft consistency, elevation reduces or eliminates the swelling
Stage II (not spontaneously reversible)	Oedema with secondary tissue changes; elevation does not eliminate the swelling
Stage III	Deforming hard swelling, partly lobular in shape, partly with typical skin changes

## Data Availability

No new data were created or analysed in this study.

## Supplementary Materials

The following supporting information can be downloaded at: https://www.mdpi.com/article/10.3390/cells14241955/s1, Table S1: Key data for inpatients ICD10:I89.0; Table S2: Key data for inpatients ICD10:I89.0; I97-2; Q82.0.
