# Supplementary material for: Definition, Epidemiology and Pathophysiology of Lymphoedema
Source: Cells. 2025 Dec 9;14(24):1955. doi: 10.3390/cells14241955 (PMC12731005; doi:10.3390/cells14241955)
Supplement: Supplementary file 1 [file cells-14-01955-s001.zip › cells-3972577-supplementary.pdf]

	Year															
	2023	2022	2021	2020	2019	2018	2017	2016	2015	2014	2013	2012	2011	2010	2005	2000
**Absolute number of cases (Germany)**																
**Total**	3238	2906	3160	3197	4627	5141	5285	5368	5207	5304	4707	4736	4265	4023	3199	2105
**Male**	1071	982	1027	1136	1434	1548	1557	1563	1512	1535	1387	1403	1260	1191	1028	574
**Female**	2167	1924	2133	2061	3193	3593	3728	3805	3695	3769	3320	3333	3005	2832	2171	1531
**Short-term inpatients (1 to 3 d)**	1011	803	813	897	1096	1194	1170	1238	1235	1196	1045	1021	942	875	717	300
**Hourly cases**	18	18	25	21	34	40	46	47	55	50	63	39	42	40	25	34
**Deaths**	.	.	.	.	.	.	.	.	.	.	.	.	.	.	.	.
**Average stay (in days)**	6.9	7.1	7.3	7.0	7.7	7.7	7.7	7.4	7.5	7.9	7.8	8.3	8.5	8.7	11.0	15.1
**The following case numbers were calculated without patients with foreign/unknown residence, age, gender.**																
**Absolute number of cases**																
**Total**	3234	2899	3150	3192	4616	5126	5261	5345	5183	5268	4695	4712	4246	4012	3178	2091
**Male**	1069	978	1024	1135	1430	1544	1546	1555	1501	1524	1379	1394	1251	1188	1017	571
**Female**	2165	1921	2126	2057	3186	3582	3715	3790	3682	3744	3316	3318	2995	2824	2161	1520
**Age-specific number of cases per 100,000 inhabitants**																
**under 15 years**	0	0	0	0	0	0	0	0	1	0	1	1	0	0	0	1
**15 to 44 years**	1	1	1	1	1	2	2	2	2	2	2	2	1	1	1	1
**45 to 64 years**	4	3	4	4	6	7	7	7	6	7	6	6	6	5	5	4
**65 years and above**	10	10	10	11	15	17	17	18	18	18	17	16	15	14	11	7
**Age-standardized number of cases per 100,000 inhabitants**																
**Total**	4	3	4	4	5	6	6	6	6	6	6	6	5	5	4	3
**Male**	2	2	2	3	3	4	4	4	4	4	3	4	3	3	3	2
**Female**	5	4	5	5	7	8	9	9	9	9	8	8	7	7	5	4

Suppl. Table S1: *Key data for inpatients ICD10:I89.0*

ICD10	Year															
	**2023**	**2022**	**2021**	**2020**	**2019**	**2018**	**2017**	**2016**	**2015**	**2014**	**2013**	**2012**	**2011**	**2010**	**2005**	**2000**
**I89.0 Lymphoedema, not elsewhere classified**	3238	2906	3160	3197	4627	5141	5285	5368	5207	5304	4707	4736	4265	4023	3199	2105
**I97.2 Postmastectomy lymphoedema syndrome**	94	81	121	80	179	205	222	245	195	217	220	201	185	198	257	373
**Q82.0 Hereditary lymphoedema**	164	11	19	27	25	20	27	20	24	12	22	33	61	67	51	63

Suppl. Table S2: *Key data for inpatients ICD10:I89.0; I97-2; Q82.0*
